# CPB-3 and CGH-1 localize to motile particles within dendrites in *C. elegans* PVD sensory neurons

**DOI:** 10.1186/s13104-021-05730-5

**Published:** 2021-08-14

**Authors:** Kathrin Spendier, Eugenia C. Olesnicky, Daniel Forand, Margaret Wolf, Darrell J. Killian

**Affiliations:** 1 Physics Department and Center for the Biofrontiers Institute, University of Colorado Colorado Springs, Colorado Springs, CO 80918 USA; 2 Department of Biology, University of Colorado Colorado Springs, Colorado Springs, CO 80918 USA; 3 Department of Molecular Biology, Colorado College, Colorado Springs, CO 80903 USA

**Keywords:** RNA-binding proteins (RBPs), CPB-3, CGH-1, Ribonucleoprotein particles (RNPs), Posttranscriptional regulation, *Caenorhabditis elegans*, Dendrites, Total internal reflection fluorescence (TIRF) microscopy

## Abstract

**Objective:**

RNA-binding proteins (RBPs) are important regulators of gene expression that influence mRNA splicing, stability, localization, transport, and translational control. In particular, RBPs play an important role in neurons, which have a complex morphology. Previously, we showed that there are many RBPs that play a conserved role in dendrite development in *Drosophila* dendritic arborization neurons and *Caenorhabditis elegans* (*C. elegans*) PVD neurons including the cytoplasmic polyadenylation element binding proteins (CPEBs), Orb in *Drosophila* and CPB-3 in *C. elegans*, and the DEAD box RNA helicases, Me31B in *Drosophila* and CGH-1 in *C. elegans*. During these studies, we observed that fluorescently-labeled CPB-3 and CGH-1 localize to cytoplasmic particles that are motile, and our research aims to further characterize these RBP-containing particles in live neurons.

**Results:**

Here we extend on previous work to show that CPB-3 and CGH-1 localize to motile particles within dendrites that move at a speed consistent with microtubule-based transport. This is consistent with a model in which CPB-3 and CGH-1 influence dendrite development through the transport and localization of their mRNA targets. Moreover, CPB-3 and CGH-1 rarely localize to the same particles suggesting that these RBPs function in discrete ribonucleoprotein particles (RNPs) that may regulate distinct mRNAs.

## Introduction

Dendrites are neuronal processes that are often highly branched such that they create a large sensory field or can receive synaptic information from many other cells. The proper development and maintenance of dendritic branches is important for sensory perception, learning, memory, and behavior, and defects in dendrite development are associated with several neurological disorders [1, 2]. Significant evidence supports the view that RNA-binding proteins (RBPs) are important for dendritic morphology across species because they can regulate several posttranscriptional mechanisms such as mRNA transport, localization, and localized translation [3, 4].

Previously, we identified a suite of conserved RBPs that play a role in regulating dendrite development in *Drosophila* larval dendrite arborization (da) neurons and *C. elegans* PVD neurons [5–10]. Among these are *Drosophila* Orb and *C. elegans* CPB-3, which are cytoplasmic polyadenylation element binding proteins (CPEBs). CPEBs have been implicated in the regulation of mRNA translation by controlling poly-(A) tail length and interacting with the translation initiation complex [11, 12]. Our previous work showed that CPB-3 localizes to particles within the soma, dendrites, and axons of PVD neurons in *C. elegans* [6]. In addition, studies in vertebrates have implicated CPEB in mRNA transport and localization within dendrites [13–15].

*Drosophila* Me31B and *C. elegans* CGH-1 have also been implicated in dendrite development and axon morphogenesis [6, 16, 17]. These RBPs are DEAD box RNA helicases related to *S. cerevisiae* Dhh1 and mammalian DDX6/RCK; broadly, DEAD box RNA helicases of this family localize to cytoplasmic ribonucleoprotein particles (RNPs), such as processing bodies (P-bodies), and have been implicated as regulators of mRNA translation and decay [18–21]. In *C. elegans*, CGH-1 localizes to particles in the soma and neurites of PVD neurons and colocalizes with mRNA decay factors within *C. elegans* touch receptor neurons where it plays a role in regulating axon regeneration and maintaining axon integrity [6, 17]. In *Drosophila* da neurons, Me31B is localized in dendrites suggesting a possible role in mRNA transport or localization, or in regulating the translation, storage, and decay of mRNAs during transit and in localized RNPs [22].

To learn more about the molecular mechanisms by which CPB-3 and CGH-1 regulate dendrite development, we expressed and observed functional fluorescent fusion proteins within PVD neurons in live *C. elegans.* We find that CPB-3 and CGH-1 localize to particles within dendrites that undergo directional movement at a speed consistent with microtubule-based active transport. A much larger proportion of CGH-1 particles displayed directed movement compared to CPB-3 particles. Of the particles exhibiting directed movement, CPB-3 particles are more likely to exhibit anterograde movement away from the soma while CGH-1 particles often move bidirectionally within dendrites. In dual labeling experiments we find that CPB-3 and CGH-1 rarely label the same particles suggesting that these RBPs may regulate different mRNAs and/or function independently of each other. Together, our results are consistent with a model in which CPB-3 and CGH-1 regulate mRNAs during their transport within dendrites, perhaps playing a critical role in regulating dendrite morphogenesis.

## Main text

### Results

#### CPB-3 and CGH-1 localize to motile particles in PVD dendrites

We previously identified CPB-3 and CGH-1 as RBPs that are important for dendrite morphogenesis of PVD neurons in *C. elegans.* Using RBP::GFP (green fluorescent protein) fusion proteins expressed in PVD neurons, we found that these RBPs localize to particles within the dendrites and soma, and we showed that these fusion proteins are functional because they rescue (albeit partially for CPB-3) the dendrite defects in mutants [6]. To better characterize the subcellular localization of CPB-3 and CGH-1 in PVD neurons, we used total internal reflection fluorescence (TIRF) microscopy (see Materials and Methods). TIRF microscopy uses an excitation laser beam that is totally internally reflected at the glass-water interface and produces an evanescent wave that travels parallel to the interface and selectively excites the sample 100 nm–1 µm from the substrate. This method greatly reduces background fluorescence and enables tracking of particles within the evanescent field [23].

We imaged a total of 147 CPB-3::GFP particles and 80 CGH-1::GFP particles. For each particle, we calculated the mean-squared displacement (MSD), plotted it as a function of time, and fit the data to the three MSD models described in Equation (Eqs. 1, 2, and 3 to categorize each particle as stationary (measured particle diffusivities below 1.0 e-3 µm^2^/s), diffusive, confined, or as making a directed run (see Materials and Methods). CPB-3 and CGH-1 particle trajectory lengths ranged from 2 to 36 s with a frame rate ($\Delta t$) of 50, 20, or 10 frames/s. Estimated particle localization uncertainty for all trajectories was determined by fitting the free, unconfined diffusion MSD Eq. (1) to the first three $2\Delta t$, $3\Delta t$, and $4\Delta t$ time points of the MSD plot. The localization uncertainty was 48 ± 21 nm for $\Delta t$ = 20 ms, 37 ± 23 nm for $\Delta t$ = 50 ms, and 34 ± 21 nm for $\Delta t$ = 100 ms where the error represents standard error of the mean (SEM). The corresponding average uncertainty in CPB-3 and CGH-1 particle localization was calculated to be 40 ± 22 nm.

**Fig. 1:**
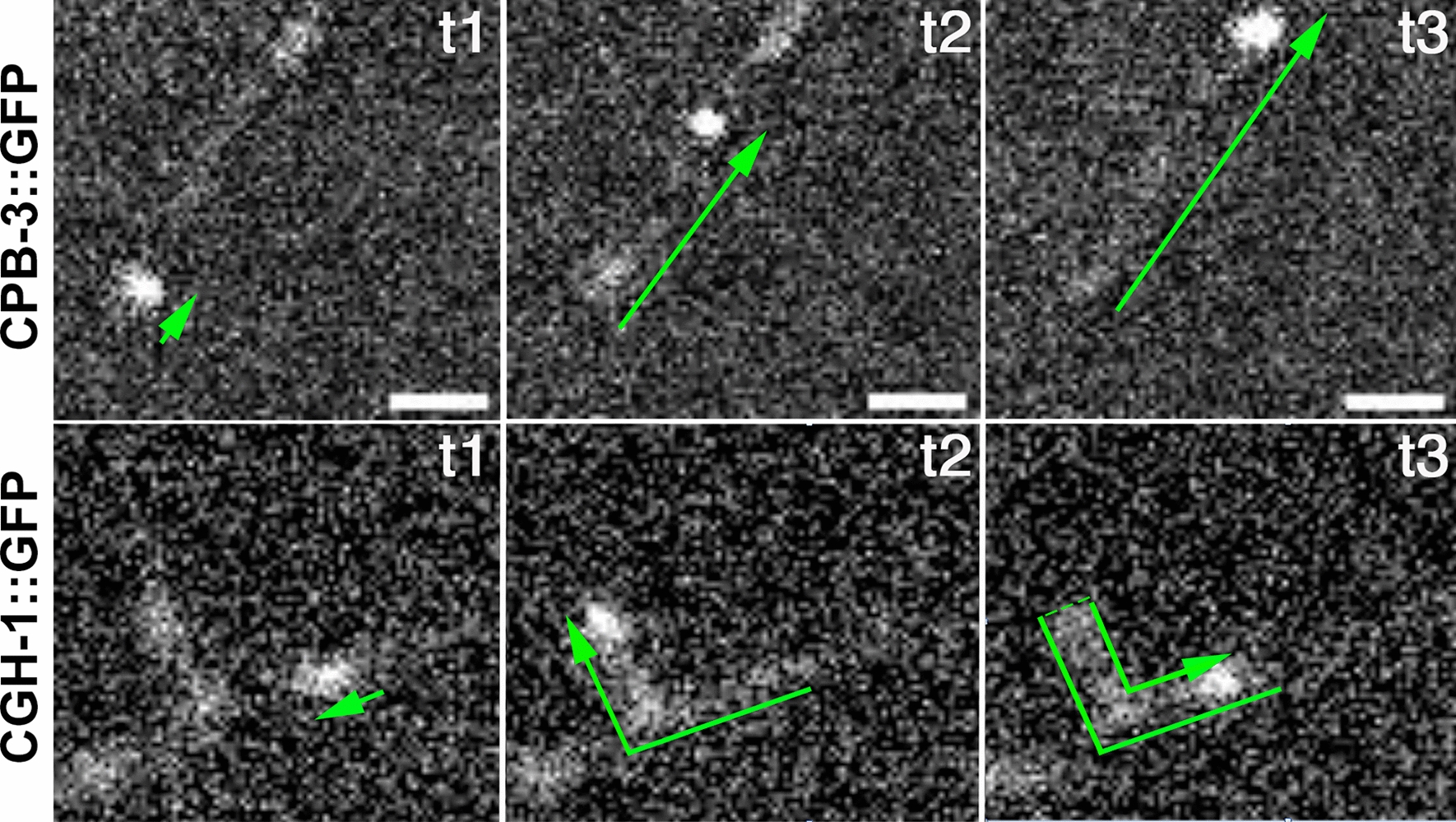
CPB-3 and CGH-1 particles are motile in PVD dendrites. Arrows outline movement over time where t1, t2, and t3 are different images from a time lapse movie with an acquisition frequency of 100 ms. Note: CGH-1 reverses direction. Bar = 1 μm

For CPB-3::GFP particles, 8% were stationary, 36% were diffusive, 48% exhibited confined movement, and 8% of particles made extended runs of directional movement. For CGH-1::GFP particles, 14% were stationary, 39% were diffusive, 1% confined (a single particle), and 46% made runs (Table 1; Fig. 1). For particles that made directed runs within dendrites, we calculated their median speed at 0.68 µm/s for CPB-3 and 0.87 µm/s for CGH-1 (Table 1). There was no statistically significant difference between CPB-3 and CGH-1 particle run speed (p = 0.9 by two-sample t-test).

**Table 1:** Parameter vales obtained from MSD fits. SEM represents standard error of the mean

Mode of transport (# of cells)	Fit Eq	# of tracks	Speed (µm/s), Median, (75^th^, 25^th^)	Diffusivity (µm^2^/s), Median, (75^th^, 25^th^)	Confinement length L_x_ (µm), Median, (75^th^, 25^th^)	Confinement length L_y_ (µm), Median, (75^th^, 25^th^)
CPB-3		**147**				
Stationary (5 cells)	Equation (1)	12		3.2 e-4 (5.4 e-4, 1.2 e-4)		
Diffusive (9 cells)	Equation (1)	53		0.0087 (0.052, 0.0023)		
Confined (10 cells)	Equation (3)	70		0.087 (0.32,0.0085)	0.61 (0.99, 0.21)	0.037 (0.082, 0.026)
Runs (7 cells)	Equation (2)	12	0.68 (1.97, 0.24)			
CGH-1		**80**				
Stationary (7 cells)	Equation (1)	11		4.1 e-4 (7.4 e-4, 2.5 e-4)		
Diffusive (11 cells)	Equation (1)	31		0.010 (0.027, 0.0044)		
Confined (1 cell)	Equation (3)	1		0.29	0.46	0.39
Runs (10 cells)	Equation (2)	37	0.87 (1.65, 0.43)

We also evaluated the direction of CPB-3 and CGH-1 particle movement within dendrites. Of 60 CPB-3::GFP particles exhibiting directed movement, 45% (*n* = 27) exhibited anterograde movement away from the soma, 18% (*n* = 11) exhibited retrograde movement, and 37% (*n* = 22) exhibited bidirectional movement. Of 52 CGH-1::GFP particles exhibiting directed movement, 23% (*n* = 12) were anterograde, 12% (*n* = 6) were retrograde, and 65% (*n* = 34) were bidirectional (see Fig. 1).

#### CPB-3 and CGH-1 mostly localize to distinct cytoplasmic particles

**Fig. 2:**
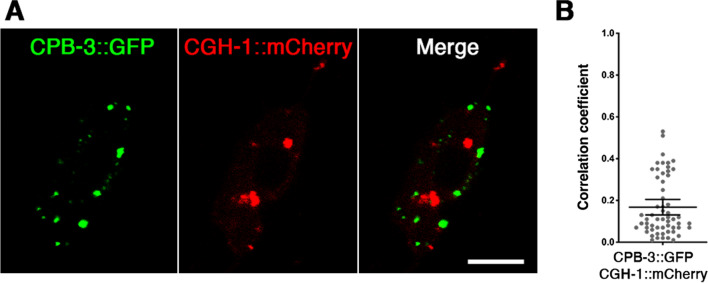
**A** CPB-3::GFP and CGH-1::mCherry label distinct particles in the cytoplasm of PVD neurons. Bar = 5 μm. **B** 58 confocal Z-stack images from 12 PVD neurons were analyzed by for colocalization using the ImageJ plug-in Coloc2 to obtain Pearson's correlation coefficients. The average correlation coefficient is 0.168; error bars represent the 95% confidence interval

To learn if CPB-3 and CGH-1 localize to the same particles, we expressed CPB-3::GFP and CGH-1::mCherry fusion proteins in PVD neurons, imaged them using confocal microscopy, and performed a colocalization analysis (see Materials and methods; Fig. 2). We found that CPB-3::GFP and CGH-1::mCherry particles have a Pearson correlation coefficient of 0.168 where 0 represents no colocalization and 1 is perfect colocalization [24]. Thus, the data suggest, at best, a weak colocalization of CBP-3 and CGH-1 particles. Furthermore, the correlation coefficient may be an overestimate given that CBP-3 and CGH-1 particles could have been trapped within the same compartment, moving around within the same confinement zone, thus appear colocalized due to their proximity and the limitations of confocal microscopy. Nonetheless, the majority of CPB-3 and CGH-1 foci do not colocalize.

### Conclusions

Here we show that CPB-3 and CGH-1 localize to cytoplasmic particles within PVD neurons, some of which are motile within dendrites. This is consistent with CPB-3 and CGH-1 regulating posttranscriptional gene expression by acting within RNPs to regulate mRNA transport, localization, stability, and localized translation. Importantly, CPB-3 and CGH-1 are likely to have evolutionarily conserved roles in regulating RNA within dendrites, as CPEB is found in motile particles in vertebrate dendrites and Me31B localizes to RNPs within dendrites in *Drosophila* [13–15, 22]. The underlying architecture of dendrites is based on microtubules and numerous studies have implicated the microtubule-based motor proteins kinesin and dynein in the transport of RNPs within dendrites [25–29]. The speed of particles with directed movement that we observed (0.68–0.87 µm/s) is consistent with observed speeds for kinesin-mediated and dynein-mediated transport within neurons (~ 1 µm/s) [30]. Importantly, we see CPB-3 and CGH-1 are mostly localized to distinct particles. This suggests that CPB-3 and CGH-1 may regulate different mRNAs and/or function independently of each other. Together, our results support a model in which CPB-3 and CGH-1 regulate mRNAs during their transport within dendrites, perhaps playing a critical role in regulating dendrite morphogenesis.

### Materials and Methods

#### *C. elegans* genetics and transgenic strains

Strains were derived from the Bristol strain N2, grown at 20C, and constructed using standard procedures [31]. For analysis of motile particles within PVD dendrites, *cpb-3* and *cgh-1* cDNAs were expressed as fusion proteins to GFP under the control of the PVD-specific promoter *ser2prom3* [6, 32]. For colocalization studies, strain DJK172: *unc-76(e911); cnjEx[s2p3::cpb-3::GFP, s2p3::cgh-1::cherry, unc-76(* +*)]* was created by injecting plasmid pDJK247 (*ser2prom3::cpb-3 cDNA::GFP*) at 20 ng/ul, pDJK293 (*ser2prom3::cgh-1 cDNA::mCherry*) at 20 ng/ul, and *unc-76(* +*)* at 60 ng/ul into *unc-76(e911)* animals. DNA microinjection was performed using standard practices [33]. For all microscopy, adult animals were mounted on coverslips with 2% agarose pads, and immobilized with 600 mM levamisole.

#### TIRF microscopy

TIRF microscopy was used to visualize CPB-3::GFP and CGH-1::GFP motion. The penetration depth of the evanescent wave for 488 nm excitation was between 600—700 nm. Objective-based TIRF microscopy was performed with a S-TIRF module (Spectral Applied Research, Canada) attached to a Leica DMI3000 B inverted microscope with a 100 × and 1.47 N.A. oil immersion objective using a 488 nm laser (Coherent Inc.). A 1.5 × lens was added to the excitation beam path resulting in a final magnification of 150x. A 550/20 nm single-band bandpass filter (Chroma) was used to collect fluorescence. TIRF images were collected at room temperature with an EMCCD camera (Evolve Delta; Photometrics) operated by Micro-Manager [34]. Image series were collected with image exposure of 20 ms, 50 ms or 100 ms depending on particle brightness.

#### Single-particle tracking

Images were processed in MATLAB (MathWorks, Inc., Natick, MA), in conjunction with the DIPImage image processing toolbox [35]. Image backgrounds were averaged and subtracted to reduce noise. Particle coordinates (x,y) were identified in each frame by a direct Gaussian fit algorithm [36, 37] and linked together into trajectories. To identify the nature of particle motion, the MSD $\langle {r}^{2}(\Delta t)\rangle$, a measure of the average (denoted by brackets) distance a molecule travels, was calculated for each time difference $\Delta t$ in the track record. After computing the MSD, a plot of square-displacements as a function of time has the ability to resolve different modes of particle motion [38]. The MSD signature for two-dimensional free, unconfined diffusion is linear.

1 $$\langle {r}^{2}(\Delta t)\rangle =offset+4D\Delta t,$$

where *D* is the particle’s diffusion coefficient and the *offset* represents the sum of the localization error [39]. For directed particle motion (runs) with speed *v*, the MSD plot exhibits an upward curvature.

2 $$\langle {r}^{2}(\Delta t)\rangle =offset+4D\Delta t+{v}^{2}\Delta {t}^{2}.$$

The third observed two-dimensional mode of particle motion, was confined diffusion with MSD described as.

3 $$\langle {r}^{2}(\Delta t)\rangle =offset+\frac{{L}_{x}^{2}}{6}\left[1-exp(-{\pi }^{2}D\Delta t/{L}_{x}^{2})\right]+\frac{{L}_{y}^{2}}{6}\left[1-exp(-{\pi }^{2}D\Delta t/{L}_{y}^{2})\right].$$

In confined diffusion the MSD graph has a downward curvature and asymptotically approaches L_x_^2^/6 and L_y_^2^/6. In this mode the particle diffuses within a limited area bounded by 0 ≤ x ≤ L_x_ and 0 ≤ y ≤ L_y_.

#### Directionality of CPB-3 and CGH-1 particles

Observation of particle direction was performed with a Leica DM500B epifluorescence microscope with a 63 × objective.

#### Colocalization of CPB-3 and CGH-1

Strain DJK247 was imaged with a Leica SP5 spectral confocal microscope at 63 × with 0.5 µm per step and Leica LAS software. Colocalization of CPB-3::GFP and CGH-1::Cherry in PVD neurons was analyzed by ImageJ plug-in Coloc2 (http://fiji.sc/Coloc2) on regions of interest corresponding to the PVD soma to obtain Pearson's correlation coefficients [10, 40].

## Limitations of the study

- To facilitate the visualization of RBP-containing particles we used a transgene expression system that likely leads to overexpression. We cannot rule out the possibility that overexpression may impact the number, size, and behavior of the RBP-containing particles.
- While we used a highly sensitive TIRF microscopy system to image RBP-containing particles, we cannot rule out the possibility that some particles were below the threshold for our methods of detection.
- Since this study was restricted to examining particles within PVD neurons in *C. elegans*, it is not clear if CPB-3 and CGH-1 localize to motile particles with similar properties in other types of neurons, or if their orthologs do so in other species.

## Data Availability

*C. elegans* strains and transgenes (plasmids) are available upon request.

## Abbreviations

RBP: RNA-binding protein
*C.** elegans*: *Caenorhabditis elegans*
CPEB: Cytoplasmic polyadenylation element binding protein
RNP: Ribonucleoprotein particle
da neuron: Dendrite arborization neuron
P-body: Processing body
TIRF: Total internal reflection microscopy
MSD: Mean-squared displacement
Eq: Equation
SEM: Standard error of the mean
GFP: Green fluorescent protein
